# Combination of transbronchoscopic oxygen insufflation and a digital chest drainage system in endobronchial occlusion: a hybrid technique for localization of fistula in intractable pneumothorax

**DOI:** 10.1186/s12890-024-03043-4

**Published:** 2024-06-05

**Authors:** Shunsuke Ueno, Toshiyuki Nakai, Yasuyuki Mizumori, Fukumichi Ishiyama, Kentaro Ueno, Manami Kouno, Yuri Oshima, Misako Nishimura, Atsushi Miyamoto, Yoshiya Matsumoto, Kenji Sawa, Kanako Sato, Kazuhiro Yamada, Tetsuya Watanabe, Kazuhisa Asai, Kenichiro Otani, Tomoya Kawaguchi

**Affiliations:** 1 Department of Respiratory Medicine, Osaka Metropolitan University, 1-4-3, Asahimachi, Abeno-Ku, Osaka-City, Osaka 545-8585 Japan; 2 Department of Respiratory Medicine, National Hospital Organization, Himeji Medical Center, 68, Honmachi, Himeji-City, Hyogo 670-8520 Japan; 3 Department of Clinical Oncology, Osaka Metropolitan University, 1-4-3, Asahimachi, Abeno- Ku, Osaka-City, Osaka 545-8585 Japan; 4 Department of Respiratory Medicine, Yodogawa Christian Hospital, 1-7-50, Kunijima, Higashi Yodogawa-Ku, Osaka-City, Osaka 533-0024 Japan

**Keywords:** Intractable pneumothorax, Prolonged air leak, Transbronchoscopic oxygen insufflation, Digital chest drainage system, Endobronchial occlusion

## Abstract

**Background:**

The management of intractable secondary pneumothorax poses a considerable challenge as it is often not indicated for surgery owing to the presence of underlying disease and poor general condition. While endobronchial occlusion has been employed as a non-surgical treatment for intractable secondary pneumothorax, its effectiveness is limited by the difficulty of locating the bronchus leading to the fistula using conventional techniques. This report details a case treated with endobronchial occlusion where the combined use of transbronchoscopic oxygen insufflation and a digital chest drainage system enabled location of the bronchus responsible for a prolonged air leak, leading to the successful treatment of intractable secondary pneumothorax.

**Case presentation:**

An 83-year-old male, previously diagnosed with chronic hypersensitivity pneumonitis and treated with long-term oxygen therapy and oral corticosteroid, was admitted due to a pneumothorax emergency. Owing to a prolonged air leak after thoracic drainage, the patient was deemed at risk of developing an intractable secondary pneumothorax. Due to his poor respiratory condition, endobronchial occlusion with silicone spigots was performed instead of surgery. The location of the bronchus leading to the fistula was unclear on CT imaging. When the bronchoscope was wedged into each subsegmental bronchus and low-flow oxygen was insufflated, a digital chest drainage system detected a significant increase of the air leak only in B5a and B5b, thus identifying the specific location of the bronchus leading to the fistula. With the occlusion of those bronchi using silicone spigots, the air leakage decreased from 200 mL/min to 20 mL/min, and the addition of an autologous blood patch enabled successful removal of the drainage tube.

**Conclusion:**

The combination of transbronchoscopic oxygen insufflation with a digital chest drainage system can enhance the therapeutic efficacy of endobronchial occlusion by addressing the problems encountered in conventional techniques, where the ability to identify the leaking bronchus is dependent on factors such as the amount of escaping air and the location of the fistula.

## Background

Pneumothorax is generally classified as spontaneous or secondary, with the latter being commonly caused by chronic obstructive pulmonary disease or interstitial lung disease. Pneumothorax is typically treated with thoracic drainage followed by pleurodesis; however, failure of these treatments leads to intractable pneumothorax with prolonged air leakage, which requires surgical closure of the fistula. The clinical problem associated with intractable secondary pneumothorax (ISP) is that those patients are often not suitable for surgery because of ineffectual respiratory conditions due to the underlying disease [1]. Consequently, ISP without surgical indications poses a considerable challenge for respiratory physicians in clinical practice, given its poor prognosis and the absence of systematic treatment strategies [2].

Endobronchial occlusion (EO) under bronchoscopy is reported to be a useful non-surgical treatment for intractable pneumothorax [3, 4], and is essential for accurate identification of bronchi responsible for fistulas to treat ISP. Conventional EO techniques use computed tomography (CT) or balloon occlusion tests to identify the bronchus responsible for the fistula. However, ISP often involves collateral ventilation and multiple fistulas due to extensive pleural damage caused by underlying disease, which makes it difficult to identify the bronchi responsible for the fistula with conventional techniques. Moreover, confirming the effect of balloon occlusion on the amount of air leakage (AAL) is affected by observer variability and difficulty detecting changes in AAL with the unaided eye. This report describes a case of ISP where we identified the bronchi responsible for the fistulas using a hybrid technique, combining transbronchoscopic oxygen insufflation with a digital chest drainage system (DCDS). This hybrid technique assisted in the successful treatment of the ISP with EO using silicone spigots.

## Case Presentation

**Fig. 1:**
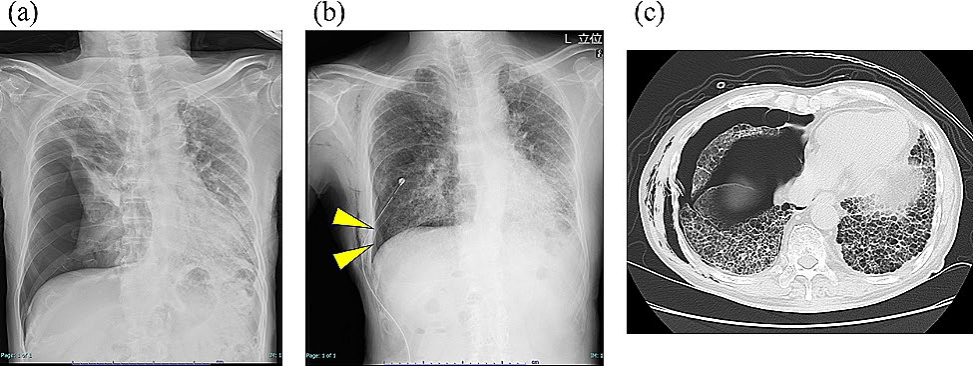
Images taken at admission and post-thoracic drainage. At the emergency visit, the chest X-ray revealed a pneumothorax in the right lung with mediastinal shift (**a**). After thoracic drainage, a chest X-ray showed a mild pneumothorax cavity (arrowhead) (**b**), while chest computed tomography revealed collapse of the right middle and lower lobes against a background of ground-glass opacities and honeycombing in both lungs (**c**)

An 83-year-old man was referred to our hospital with sudden chest pain and dyspnea. He had been diagnosed with chronic hypersensitivity pneumonitis (CHP) 3 years previously and had been receiving treatment with oral corticosteroid (5 mg/day) and home oxygen therapy since then. A chest radiograph showed a right pneumothorax with mediastinal shift, diagnosed as a secondary pneumothorax associated with the advance state of CHP. A 20 F thoracic drainage tube was placed (Fig. 1), but a chest radiograph showed incomplete expansion of the right lung, and marked air bubbling into the analog chest drainage system during expiration persisted, corresponding to Cerfolio classification, Grade 2 [5]. The right upper lobe was fully expanded, and the presence of bronchopleural fistulas in the middle or lower lobes was indicated by chest CT performed after placing the drainage tube, but the precise locations were unclear. The air leak persisted for 5 days, and the pneumothorax was considered intractable. The chest drainage system was changed from analog to digital (Thopaz®, Medela, Baar, Switzerland) for the accurate measurement of the air leakage. Surgery to close the fistulas under general anesthesia was deemed unsafe due to poor pulmonary function and general condition. Therefore, we performed EO using silicone spigots (Novatech, La Ciotat, France) on day 10 of the hospital stay.

**Fig. 2:**
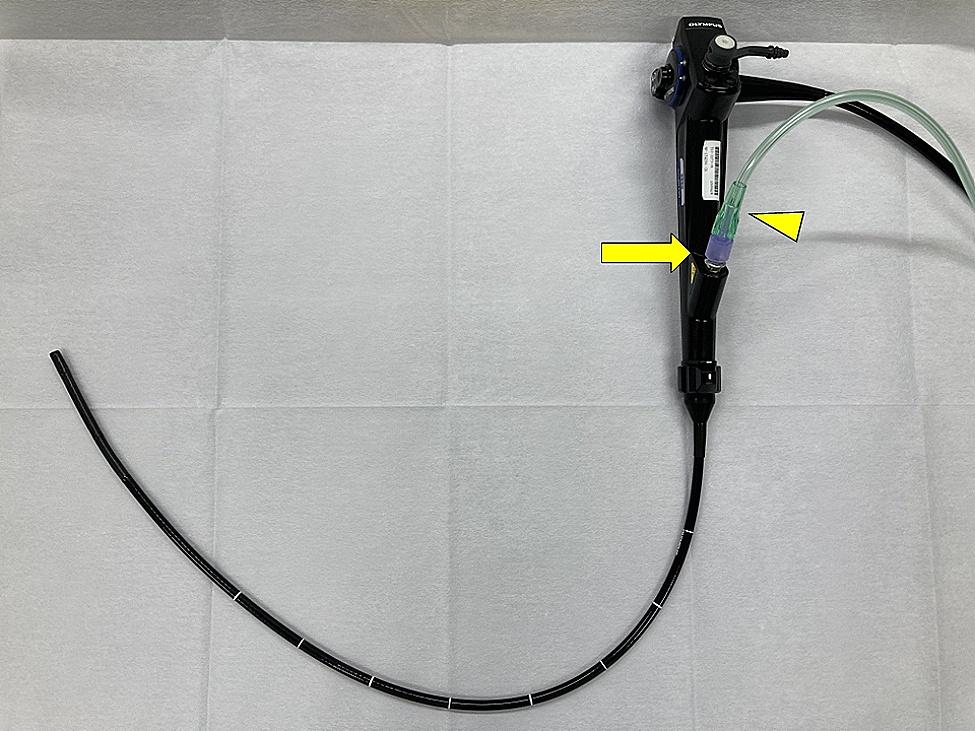
Equipment for transbronchoscopic oxygen insufflation. Oxygen tubing (001503, Next Japan Medicalnext Co., Ltd., Osaka, Japan) (arrowhead) connected to the working channel of the thick bronchoscope (BF-1TQ290, Olympus, Tokyo, Japan) using a connector (MM 04617, Top, Tokyo, Japan) (arrow). After wedging the bronchoscope tip into the subsegmental bronchus, transbronchoscopic oxygen insufflation was performed by with oxygen at the low flow rate of 2 L/min

**Fig. 3:**
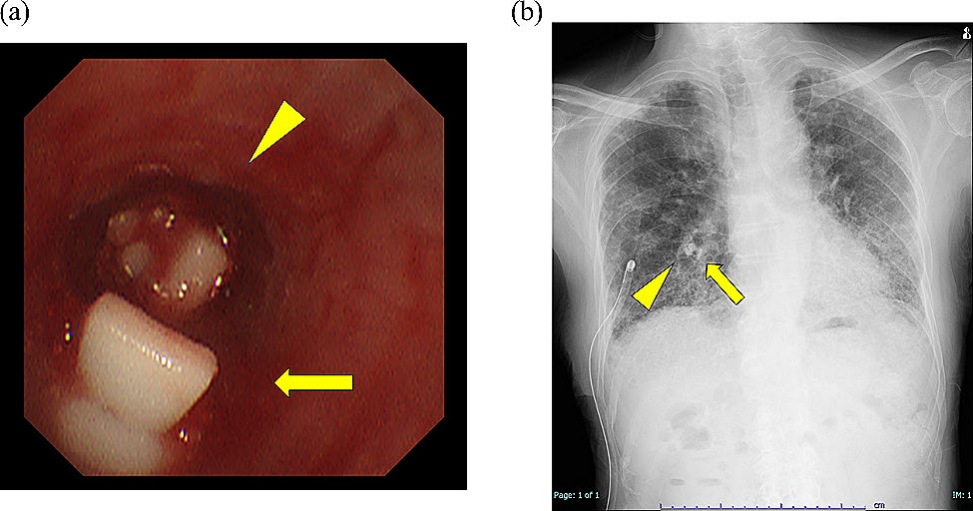
Endobronchial occlusion with silicone spigots. The combination of transbronchoscopic oxygen insufflation and a digital chest drainage system identified B5a and B5b as the bronchi responsible for the fistulas. B5a and B5b were occluded with size M (arrowhead) and S (arrow) spigots, respectively, and consequently the amount of air leakage decreased significantly (**a**: bronchoscopy image) (**b**: chest radiograph)

The EO procedure was performed using flexible bronchoscopes (thick scope BF-1TQ290 and thin scope BF-P290, Olympus, Tokyo, Japan) under local anesthesia with a combination of fentanyl and midazolam for conscious sedation. The patient underwent fiberoptic intubation with an 8.5-mm uncuffed endotracheal tube (Portex® Siliconised PVC Oral/Nasal Uncuffed Tracheal Tube, Smiths Medical, Minneapolis, MN, USA). The AAL, initially measured at 100 mL/min in the sitting position, decreased to approximately 20 mL/min when the patient assumed a supine position. To locate the fistula, oxygen was insufflated at a low flow rate (2 L/min) for 15 to 30 s through the working channel of the thick bronchoscope after wedging the bronchoscope tip sequentially into each subsegmental bronchus in the right middle and lower lobe, and the AAL was measured with a DCDS (Fig. 2). Increased AAL was observed when oxygen was insufflated into B5a and B5b (from 20 mL/min to approximately 200 mL/min in both bronchi), but not the other subsegmental bronchi. Therefore, we judged that there was a bronchopleural fistula on the peripheral side of B5a and B5b. These bronchi were occluded by inserting spigots (size M, 6-mm diameter for B5a, and size S, 5-mm diameter for B5b) using a thin bronchoscope (Fig. 3). After bronchial occlusion, the AAL on the DCDS decreased significantly to ~ 0, so EO was terminated.

The following day, an autologous blood patch (ABP) was performed because a minor pneumothorax in the right lung was visible on chest radiograph and a slight air leak was observed when the patient was asked to cough. The air leak completely disappeared after ABP, and the thoracic drainage tube was removed. On day 17 of hospitalization, the patient was discharged.

## **Discussion and conclusions**

A problem associated with conventional EO for ISP is the difficulty in locating the fistula causing the air leak. In this case, the combination of transbronchoscopic oxygen　insufflation and a DCDS enabled accurate measurement of oxygen leak through the fistula and precise identification of the bronchi responsible for the fistula, resulting in the successful treatment of the ISP by EO. This hybrid technique is easily implementable in clinical practice and can potentially assist EO for ISP.

While balloon occlusion is commonly used for identifying bronchopulmonary fistulas during EO, one problem with this technique is that wedging the balloon catheter into subsegmental bronchi is difficult depending on their positions and branching angle. Moreover, temporary disappearance of the air leak during the procedure, contingent upon the AAL and position of the fistula, introduces uncertainty in leak detection with this technique [6]. A simple and accurate technique to locate fistulas is needed. The transbronchoscopic oxygen insufflation performed in this case utilizes the flexibility of the bronchoscope to occlude the subsegmental bronchus by itself more easily than the balloon technique. Low flow oxygen through the fistula may facilitate the quick localization of the bronchus contributing to the prolonged air leak, irrespective of the AAL or location of the bronchopulmonary fistula. The risk of iatrogenic barotrauma or worsening air leakage associated with oxygen inflow, which is a concern, can be minimized by considering the actual oxygen dose during the procedure [7]. However, prolonged oxygen insufflation can also result in other adverse events such as tension pneumothorax and air embolism. These risks may be reduced by minimizing the duration of oxygen insufflation and allowing breaks before the insufflation of each subsegmental bronchus.

Another noteworthy aspect in this case is the use of a DCDS to detect air leaks. The conventional analog system has challenges in detecting changes in intraoperative leakage volume when air leaks are intermittent and low volume or, conversely, when the AAL is too high. Furthermore, ISP associated with interstitial pneumonia often has several subsegmental bronchi connected to fistulas due to collateral ventilation between lung subsegments; therefore, even if one bronchus is occluded with a balloon, it was difficult for the physician to confirm its effect on the AAL by visualizing air bubbles [8]. In contrast, a DCDS quantifies these changes for the physician and, when combined with transbronchoscopic oxygen insufflation, can be a more effective technique for locating fistulas. A DCDS can also graph the AAL over time and has been reported to facilitate the early removal of drainage tubes in cases of pneumothorax or video-assisted thoracoscopic lobectomy [9, 10]. The application of DCDS immediately after chest drainage in patients with risk factors for persistent air leaks, such as interstitial lung disease, chronic obstructive pulmonary disease, or low body mass index, may enable early prediction of the development of intractable pneumothorax.

Pleural reinforcement from outside the fistula is required when an air leak is not stopped by EO alone. In this case, an air leak with a volume of approximately 20 mL/h remained after EO, just at the recommended volume for safely removing a drainage tube [1]. However, given the high risk of recurrence of pneumothorax due to the advanced state of CHP and chronic steroid use, an ABP was added to seal the fistulas from the pleural exterior. Accordingly, the air leak disappeared completely following application of the ABP, and the drainage tube was safely removed. Although the necessity and advantage of additional ABP against minor leaks after EO remains to be unestablished, there are reports of a high success rate (89%) for ABPs in stopping prolonged air leaks after pulmonary surgery with minimal impact on lung function and milder adverse events than other chemical pleurodesis [11, 12]. Another advantage of ABPs is that they stop air leakage by patching the fistula rather than by adhesion between the pleura; thus, they can be effective even if full lung expansion is not achieved after thoracic drainage [13]. In cases of ISPs, minor air leaks and incomplete lung expansion attributed to pleural damage and low pleural compliance often remain, despite the application of EO. The addition of an ABP could safely and effectively complement the treatment of EO for ISP.

In conclusion, the hybrid technique combining transbronchoscopic oxygen insufflation and DCDS enables the precise detection of the bronchi responsible for fistulas and assists EO in treating ISP.

## Data Availability

All data generated or analyzed during this study are included in this article. Further enquiries can be directed to the corresponding author.

## Abbreviations

ISP: Intractable secondary pneumothorax
EO: Endobronchial occlusion
CT: Computed tomography
AAL: Amount of air leakage
DCDS: Digital chest drainage system
ABP: Autologous blood patch
CHP: Chronic hypersensitivity pneumonitis
